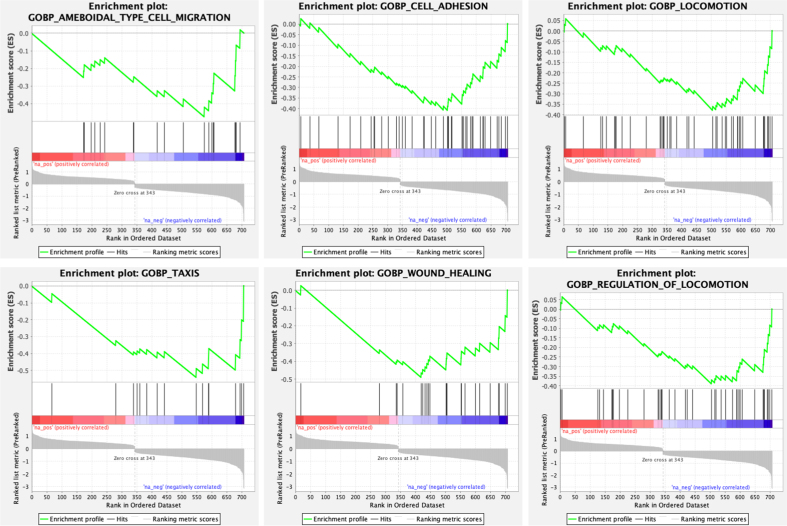# Spatial distribution and functional relevance of FGFR1 and FGFR2 expression for glioblastoma tumor invasion

**DOI:** 10.1016/j.canlet.2023.216349

**Published:** 2023-09-01

**Authors:** Nawal Alshahrany, Ayesha Begum, Dorit Siebzehnrubl, Ana Jimenez-Pascual, Florian A. Siebzehnrubl

**Affiliations:** a Cardiff University School of Biosciences, European Cancer Stem Cell Research Institute, Cardiff, CF24 4HQ, United Kingdom; b Cardiff University School of Pharmacy and Pharmaceutical Sciences, Cardiff, CF10 3NB, United Kingdom

**Keywords:** Fibroblast growth factor, Migration, Xenograft, RNA sequencing, Cancer stem cell

## Abstract

Glioblastoma is the most lethal brain cancer in adults. These incurable tumors are characterized by profound heterogeneity, therapy resistance, and diffuse infiltration. These traits have been linked to cancer stem cells, which are important for glioblastoma tumor progression and recurrence. The fibroblast growth factor receptor 1 (FGFR1) signaling pathway is a known regulator of therapy resistance and cancer stemness in glioblastoma. FGFR1 expression shows intertumoral heterogeneity and higher FGFR1 expression is associated with a significantly poorer survival in glioblastoma patients. The role of FGFR1 in tumor invasion has been studied in many cancers, but whether and how FGFR1 mediates glioblastoma invasion remains to be determined. Here, we investigated the distribution and functional relevance of FGFR1 and FGFR2 in human glioblastoma xenograft models. We found FGFR1, but not FGFR2, expressed in invasive glioblastoma cells. Loss of FGFR1, but not FGFR2, significantly reduced cell migration *in vitro* and tumor invasion in human glioblastoma xenografts. Comparative analysis of RNA-sequencing data of FGFR1 and FGFR2 knockdown glioblastoma cells revealed a FGFR1-specific gene regulatory network associated with tumor invasion. Our study reveals new gene candidates linked to FGFR1-mediated glioblastoma invasion.

## Highlights

• We find that FGFR1 is present on invasive glioblastoma cells, whereas FGFR2 is absent in these cells and only expressed in the tumor mass.
• Genetic ablation of FGFR1 in patient-derived glioblastoma cell lines confirms functional relevance of FGFR1 for glioblastoma cell migration and invasion.
• By contrast, knockdown of FGFR2 had no effect on glioblastoma cell migration or tumor invasion.
• Comparative RNA sequencing analysis of control, FGFR1 and FGFR2 knockdown cells revealed gene-regulatory networks relevant for tumor invasion that are specifically associated with FGFR1.

## 1 Introduction

Glioblastoma (GBM) is the most common and malignant type of brain tumor in adults [1]. Histopathological hallmarks of GBM include diffuse infiltration of cancer cells, high mitotic activity, vascular proliferation, and pseudopalisading necrosis [2]. GBM accounts for 50% of gliomas and most frequently presents in patients aged 40–60, with a higher incidence in men than women [3].

Current standard of care includes maximum safe surgical resection followed by radiotherapy and temozolomide chemotherapy [[3], [4], [5]]. With therapy, the median survival of GBM patients is only 9–18 months from the time of diagnosis because recurrent disease develops within 6–9 months after treatment making clinical management challenging [3,6]. The causes of GBM recurrence remain unclear, however, it has been suggested that the infiltrative nature of GBM cells, intratumoral heterogeneity, and the presence of GBM cancer stem cells (GSC) [7,8] contribute to recurrence. Recent precision medicine approaches including tyrosine kinase inhibitors and immunotherapy showed some promising results for treating GBM patients, underlining the need to understand GBM biology for the development of better therapies.

GSCs constitute a small population of tumor cells that can initiate and drive tumor development. GSCs are more resistant to chemo- and radiotherapy and more invasive than other cells of the same tumor [9,10]. GSCs can manipulate their microenvironment to promote self-renewal and escape checkpoints of differentiation [7,11,12]. Several signaling pathways regulating stemness and proliferation similar to neural stem cells have been identified in GSCs including Sonic hedgehog, Notch, and Wnt [12,13]. Furthermore, GSCs express transcription factors similar to neural stem cells including OLIG2, ZEB1, and SOX2 [9,11,14]. The transcription factor ZEB1 is a key regulator of GBM stemness, tumor invasion and therapy resistance [9]. In hypoxic areas, the HIF1α-ZEB1 axis mediates a hypoxia-induced mesenchymal shift in GBM cells, resulting in enhanced stemness, tumorigenicity and decreased patient survival [15,16]. SOX2 is a well-established transcriptional regulator of GSCs, which is regulated through direct and indirect effects of ZEB1 [9,14]. SOX2 is overexpressed in 90% of GBM patient samples and in undifferentiated GSCs, supporting its relevance for maintaining GSC stemness. Moreover, SOX2 silencing causes proliferation impairment, reduction in tumor growth and invasion and cell cycle arrest [17].

Many studies have established the critical role of receptor tyrosine kinases in GBM, including fibroblast growth factor receptors (FGFRs). For example, overexpression of FGFR1, specifically in the form of FGFR1-β, has been detected in GBM compared to normal levels of the receptor in the white matter [18]. FGFR1 expression increases with WHO grade in astrocytomas, and it has been used as a marker for poor prognosis [19,20]. In U251 cells, FGFR1 expression promotes tumor growth and invasion via AKT/MAPK and RAC1/CDC42 pathways, respectively [21]. Moreover, FGFR1 point mutations (N546K and R576W) in the tyrosine kinase domain contribute to GBM growth due to enhanced protein-protein interactions and an increased likelihood of FGFR1 autophosphorylation [22]. By contrast, FGFR2 expression in the white matter is abundant, compared to malignant astrocytomas in which it is barely detectable [18]. Also, *FGFR2* is considered a GBM-associated tumor suppressor gene and high expression of FGFR2 in GBM is associated with increased patient survival [23,24]. Low FGFR2 expression in GBM is attributed to the loss of heterozygosity of chromosome 10 found in 80% of GBMs, where the FGFR2 gene is localized (10q26) [19,24]. Chromosomal translocations can cause oncogenic fusion between FGFR1/3 tyrosine kinase domains and the transforming acidic coiled-coil (TACC) domains of TACC1/3 respectively. This results in FGFR autophosphorylation and constitutive kinase activity. While FGFR1-TACC1 fusions are more associated with low-grade neuroepithelial tumors [25], FGFR3-TACC3 fusions occur in 3–7% of GBM, where they promote malignancy [26,27].

Others and we have demonstrated that FGFR1 is expressed on GSCs, with FGF2 binding to FGFR1 activating downstream signaling pathways that maintain cancer stemness [11,28,29]. Furthermore, the transcription factor ZEB1 regulates FGFR1 expression, thereby closing a positive feedback loop.

Here, we investigate the regional expression of FGFRs within the GBM tumor microenvironment and their association with GSCs. We quantify relative expression levels of FGFR1 and FGFR2 in GBM patient-derived xenograft models and compare expression within the tumor core and the invasion front.

## 2 Materials and methods

### 2.1 Cell lines

Primary human GBM cell lines L0, L1, and L2 were cultured as described previously [11]. Briefly, cells were expanded in N2 medium containing 20 ng/ml EGF and 2% bovine serum albumin, passaged every 7 days and plated at 50,000 cells/ml. Lentiviral knockdown of FGFR1 and FGFR2 was performed as described [11] and confirmed by Western blot. For immunofluorescence imaging, tumor spheres were plated onto ploy-d-lysine/laminin coated coverslips and fixed using 4% formalin solution after 24 h. Established human GBM cell lines A172, T98G, U373, and U87-MG were cultured in DMEM supplemented with 10% fetal bovine serum (FBS, Thermo Fisher) and antibiotic/antimycotic (Thermo Fisher). Confluent cells were detached using Accumax (Thermo Fisher) and plated at a density of 100,000 cells/ml (T98G, U373, U87-MG) or 300,000 cells/ml (A172).

### 2.2 Cell migration assays

For quantification of cell migration of primary human GBM lines, tumor-spheres of approximately 100–150 μm diameter were plated at low density onto poly-d-lysine/laminin coated substrates. The same spheres were imaged at 2 h and 24 h after plating. Only spheres with a diameter greater than 50 μm, 2 h after plating, were used to measure migration distance. Cell migration of established human GBM cell lines was quantified using a scratch assay. Cells were plated at 400,000–800,000 cells/ml and a scratch was made with a pipette tip 24 h after plating. Migrating cells were imaged at 0 h and 24 h.

### 2.3 Xenograft models

All animal experiments were carried out in accordance with UK Home Office regulations and the Animals (Scientific Procedures) Act 1986 (Home Office license PPL 30/3331). Mice were group-housed in 12-h light/dark cycles in filter top cages with access to food and water *ad libitum*. Cages were cleaned weekly, and nesting material as well as plastic tunnels were provided for environmental enrichment. Intracranial implantation of GBM cells as performed as described in Ref. [11]. Briefly, human GBM L2 cells [9] transduced with control, FGFR1 or FGFR2 knockdown lentiviral vectors carrying a GFP reporter were FACS purified prior to implantation. 50,000 cells were delivered in a total volume of 5 μl into the right hemisphere of female adult immunocompromised mice (n = 3 per group) [11]. Tumors were allowed to grow until the animals reached defined endpoint criteria, including body weight loss and/or neurological symptoms. Mice at endpoint were euthanized and transcardially perfused, the brains harvested, postfixed, embedded in optimal cutting temperature medium (OCT) and frozen. 30 μm coronal sections were cut on a cryostat and used for immunostaining.

### 2.4 Western blot

Protein lysates were extracted from GBM cells using RIPA buffer [11]. Protein concentration was determined using a Bradford assay. Protein lysates were diluted with Laemmli buffer (Sigma Aldrich) and denatured at 55 °C for 5 min. Proteins were separated using sodium dodecyl sulfate polyacrylamide gel electrophoresis (BioRad), transferred onto PVDF membranes using the Trans-Blot® TurboTM Transfer System (BioRad). Transferred membranes were washed in tris-buffered saline (TBS) for 10 min and blocked in 5% non-fat dry milk or bovine serum albumin in TBS-Tween (TBS-T) for 1 h, followed by two washes in TBS-T for 10 min. The membrane was incubated with primary antibodies diluted in blocking solution (Table S1) overnight at 4 °C using gentle agitation. The next day, the membrane was washed 3× in TBS-T (5 min each) and incubated with secondary antibodies for 1 h at room temperature with gentle agitation. Proteins were visualized using Clarity ECL substrate (BioRad) on a ChemiDoc Imaging System (BioRad).

### 2.5 Quantitative PCR

RNA was extracted from GBM cells using the RNeasy kit (Qiagen) and DNase digestion. cDNA was synthesized using the QuantiTect Reverse Transcription kit (Qiagen). Quantitative PCR was performed using the Takyon low Rox probe master mix dTTP blue kit (Eurogentec) and TaqMan assays for FGFR1, FGFR2, and 18S RNA as reference gene (Thermo Fisher) on a QuantStudio 7 Flex PCR system (Thermo Fisher). C_t_ values of target genes were normalized to the reference gene for each sample, and relative expression levels were determined using the delta C_t_ method.

### 2.6 Immunofluorescence staining

Tissue sections or plated tumor spheres were washed twice for 10 min each in PBS-T (PBS containing 0.1% Triton X-100). Sections were then blocked in FSB-T (Fish Skin Gelatin buffer containing 0.1% Triton X-100, 1% bovine serum albumin, and 0.2% Teleostean gelatin [9]) for 1 h at room temperature on a shaker. Sections were incubated in primary antibodies diluted in FSB-T overnight at 4 °C (Table S1). Sections were washed 3× in PBS-T (10 min each) and subsequently incubated with secondary antibodies diluted in FSB-T for 3 h at room temperature in the dark on a shaker. Nuclear staining was performed using Hoechst 33342 (Thermo Fisher) for 5 min. Sections were then washed 3× in PBS-T (10 min each), mounted on microscopic slides, and coverslipped using ProLong Diamond Antifade Mountant (Thermo Fisher).

### 2.7 Microscopy and image processing

Slides were visualized using a confocal microscope (Zeiss LSM710). Images were obtained using ZEN software. Images were processed using ImageJ software (https://imagej.nih.gov/ij/) for measuring of the mean fluorescence intensity of the core and the invasion front of the tumor (n > 14 visual fields per region).

The invasion index was calculated as described in Ref. [9]. Briefly, sequential coronal sections were picked from each brain in 360 μm intervals. All sections were imaged and images from sections containing tumor were inverted and a threshold applied to convert to binary images. The invasion index was quantified as the tumor area divided by the squared circumference.

For cell migration analysis, images were obtained on a Leica DM IL microscope equipped with a DFC3000G camera and Leica Application Suite X software and cell migration was quantified using ImageJ. For sphere assays, migration distance was calculated as the difference in sphere diameter between the two time points. For scratch assays, migration distance was calculated as the difference in gap between the two time points.

### 2.8 RNA sequencing and data processing

Total RNA was extracted from human GBM cells using the RNeasy kit (Qiagen). RNA quality was assessed on a Bioanalyzer (Agilent) with all RNA integrity (RIN) values 8 or greater. Library preparation for RNA sequencing was performed using the Ion Total RNA-Seq V2 kit and Ion Xpress RNA-Seq Barcode kit (Thermo Fisher). Library quality control was performed using the Ion Library TaqMan Quantitation kit (Thermo Fisher). RNA sequencing was run on an Ion Chef System using the Ion PI HiQ Chef kit and Ion PI chips (Thermo Fisher).

Trimming and quality control of raw sequencing data was performed using FastQC, followed by read mapping using STAR [30]. The read depth was at least 10 million mapped reads per sample. Raw read counts and RPKM values were calculated for individual exons and transcripts using an in-house script at Wales Gene Park. Differentially expressed genes were identified using DEseq2 [31]. The resultant p-values were corrected for multiple testing and false discovery issues using the FDR method [32]. Heatmaps were generated using R package clusterProfiler [33].

For analysis of publicly available RNA-seq datasets, differentially expressed gene sets were downloaded from the Allen Brain Institute and NCBI's gene expression omnibus, and z-scores or read counts for FGFR1 and FGFR2 were visualized using standard packages in R.

### 2.9 Ingenuity pathway analysis

Data were analyzed using QIAGEN Ingenuity Pathway Analysis. Data sets containing gene identifiers and corresponding data measurement values were uploaded into the application. Each identifier was mapped to its corresponding object in QIAGEN's Knowledge Base. An expression p value cutoff of 0.05 was set to identify molecules whose expression (or phosphorylation) was significantly perturbed. These molecules, called Network Eligible molecules, were overlaid onto a global molecular network developed from information contained in the QIAGEN Knowledge Base. Networks of Network Eligible Molecules were then algorithmically generated based on their connectivity.

The following analyzes were performed: canonical pathways analysis, diseases & functions, and functional network analysis. These identified the pathways from the QIAGEN Ingenuity Pathway Analysis library of canonical pathways, the biological functions, or molecules in the network that were most significant to the data set. For each analysis, molecules from the data set that met the expression p value cutoff of 0.05 and were associated with a relevant entry in the QIAGEN Knowledge Base were considered for the analysis. A right-tailed Fisher's Exact Test was used to calculate a p-value determining the probability that each canonical pathway, biological function and/or disease assigned to that data set is due to chance alone. Additionally, the significance of the association between the data set and the canonical pathway was measured by a ratio of the number of molecules from the data set that map to the pathway divided by the total number of molecules that map to the canonical pathway is displayed.

### 2.10 Statistical analysis

Statistical analyses were performed using GraphPad Prism Version 10. Normally distributed data was analyzed using T test (2 groups) or one-way ANOVA with Bonferroni post-test (3 groups). The Mann-Whitney test (2 groups) or Kruskal Wallis test (3 groups) was applied where data were not normally distributed. A p-value less than 0.05 was considered significant.

### 2.11 Data availability

RNA sequencing datasets from control and FGFR1 or FGFR2 knockdown GBM cells have been deposited in ArrayExpress (E-MTAB-13161). Other datasets analyzed in this study are available at the Allen Brain Institute (glioblastoma.alleninstitute.org) [34] and at NCBI's gene expression omnibus (GSE174470).

## 3 Results

FGFR1 and FGFR2 show an inverse relationship with GBM malignancy. Whereas high expression of FGFR1 is associated with poorer patient survival, higher expression of FGFR2 conveys a better prognosis [19,20,24]. Nevertheless, we have previously found that primary patient-derived GBM cells express FGFR2 *in vitro* [11]. Therefore, we wanted to elucidate if FGFR1 and FGFR2 are expressed in GBM *in vivo* and if there are receptor-specific patterns of expression, specifically comparing tumor core and invasive areas. We chose to determine FGFR protein expression in patient-derived xenografts, in order to visualize the core of the tumor mass as well as areas of tumor invasion within the same tissue section in these samples.

### 3.1 Validation of anti-FGFR antibodies

We first validated expression of FGFR1 and FGFR2 in 3 primary patient-derived GBM cell lines by Western blot (Fig. 1A). Comparison of GBM cells transduced with non-targeting (scrambled), shFGFR1 or shFGFR2 constructs demonstrated specificity of FGFR antibodies and confirmed that FGFR-targeting constructs ablated the targeted receptor without blocking the non-targeted receptor. We compared 2 different monoclonal FGFR1 antibodies (clone M17A3 and D8E4) for validation and excluded clone M17A3 as there were no bands visible on the Western blot (Fig. S1A). We noted that the expression levels of non-targeted receptors were closest to the control levels in GBM line L2, which we used for subsequent *in vivo* studies. Next, we used immunofluorescence staining of orthotopic xenografts of control, as well as FGFR1 and FGFR2 knockdown cells. We validated absence of FGFR1 immunostaining in xenografts of shFGFR1 cells compared to controls, as well as lack of FGFR2 staining in shFGFR2 xenografts (Fig. 1B and C). In both cases, we used human-specific anti-Vimentin or anti-Nestin antibodies to label xenografted GBM cells.

**Fig. 1:**
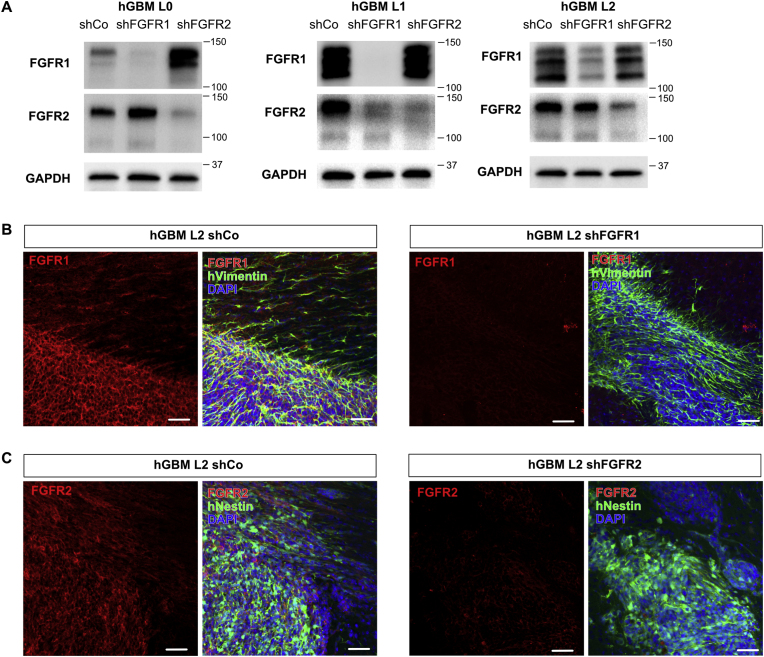
**Expression of FGFR1 and FGFR2 in GBM xenografts. (A)** Western blot of hGBM lines L0, L1, and L2 shows FGFR1-specific bands which are not present in FGFR1 knockdown cells (shFGFR1) and FGFR2-specific bands which are absent in FGFR2 knockdown cells (shFGFR2) compared to non-targeting controls (shCo). **(B)** FGFR1 immunofluorescence staining of GBM xenografts shows a specific signal that overlaps with human-specific Vimentin in tumor cells, while FGFR1 knockdown cells show no signal. Scale bar 80 μm. **(C)** FGFR2 immunofluorescence staining of GBM xenografts shows a specific signal that overlaps with human specific Nestin in tumor cells, while FGFR2 knockdown cells show no signal. Scale bars 50 μm.

### 3.2 Spatial distribution of FGFR1 and FGFR2 in xenografted GBM

**Fig. 2:**
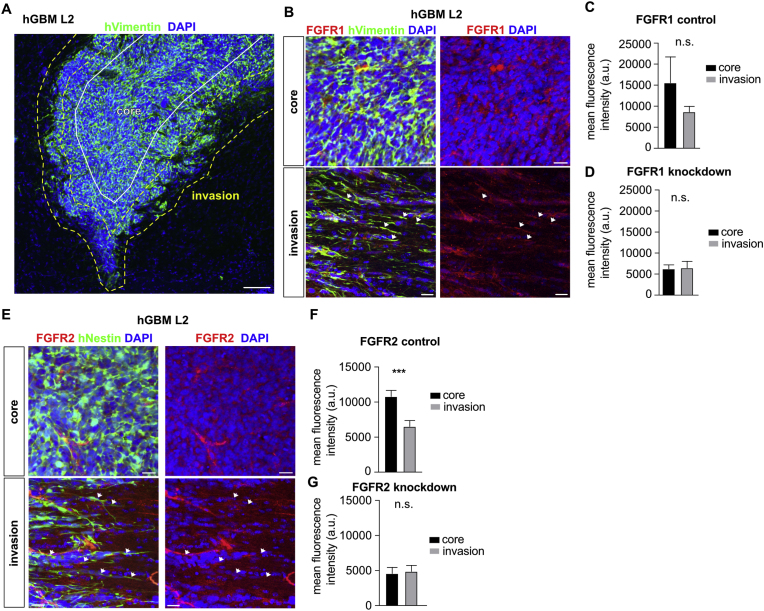
**Spatial distribution of FGFR1 and FGFR2 in GBM xenografts. (A)** Overview image showing hGBM L2 xenograft in a mouse brain with highlighted areas of tumor core (white) and invasive margin (yellow). Scale bar 100 μm. **(B)** GBM xenograft immunofluorescence staining shows expression FGFR1 in the tumor core (top panels) and invasive cells (bottom panels, arrows indicate co-staining of diffusely infiltrating cells). Scale bars 20 μm. **(C)** Quantification of mean fluorescence intensity for FGFR1 in tumor core and invasive areas shows no significant difference (n = 3 animals). **(D)** Quantification of mean fluorescence intensity for FGFR1 in xenografted FGFR1 knockdown cells shows that the fluorescence signal is lower than in control cells and no difference between tumor areas (n = 3 animals). **(E)** Immunofluorescence staining for FGFR2 in GBM xenografts shows strong staining in the tumor core (top panels), which is absent in invasive cells (bottom panels, arrows indicate diffusely infiltrating cells negative for FGFR2). Scale bars 20 μm. **(F)** Quantification of mean fluorescence intensity for FGFR2 shows significant difference between tumor core and invasive areas (n = 3 animals). **(G)** Quantification of mean fluorescence intensity for FGFR2 in xenografted FGFR2 knockdown cells shows that the fluorescence signal is lower than in control cells and no difference between tumor areas (n = 3 animals). (n.s. not significant; ***p < 0.001).

After validating anti-FGFR antibodies, we determined expression of FGFR1 in orthotopic patient-derived xenograft models of GBM, comparing central areas of the tumor core to the invasive edge (Fig. 2A). We used coronal sections from three recipient mice containing xenografted tumors and quantified mean fluorescence intensities across multiple visual fields (n > 14 per brain and region) that were either classified as tumor core (containing a mass of tumor cells) or invasive edge (containing infiltrating tumor cells intermixed with host cells). We also quantified mean fluorescence intensity from sections of mice xenografted with shFGFR1 tumors as negative controls. There was no significant difference between fluorescence intensities in the tumor core and the invasive edge, confirming visual observations that FGFR1 is expressed throughout the tumor (Fig. 2B and C). Decreased FGFR1 expression in shFGFR1 cells resulted in lower fluorescence intensity of FGFR1 staining in xenografted tumors, with no appreciable difference between tumor core and invasive edge (Fig. 2D).

We next evaluated the expression patterns of FGFR2 in GBM xenografts, again focusing on potential differences between the tumor core and the invasive edge. We quantified mean fluorescence intensities of xenografted tumors, using xenografted shFGFR2 GBM cells as negative controls. While FGFR2 was detectable in tumor core and invasive areas, we observed expression of FGFR2 on GBM cells in the tumor core, but not invasion areas (Fig. 2E, arrows). Additionally, FGFR2 mean fluorescence intensity was significantly higher in tumor core areas compared to invasive areas (Fig. 2F). As expected, ablation of FGFR2 resulted in low fluorescence intensity of FGFR2 staining (Fig. 2G). Thus, FGFR1 is expressed in both core and invasive areas of xenografted human GBM and FGFR2 is mainly expressed in the tumor core.

### 3.3 Knockdown of FGFR1 reduces tumor invasion, but FGFR2 knockdown does not

**Fig. 3:**
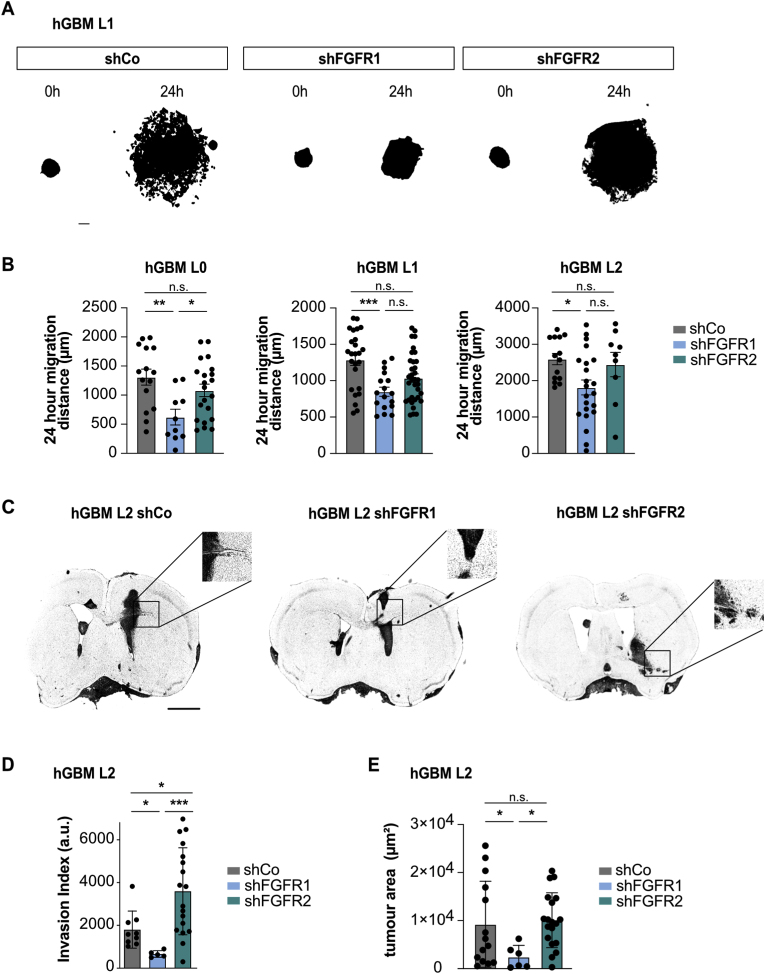
**FGFR1, but not FGFR2, regulates GBM cell migration and tumor invasion. (A)** Representative threshold images of plated tumor spheres used for quantification of migration. Scale bar 200 μm. **(B)** Quantification of cell migration distances (sphere outgrowth assay) for three different primary patient derived GBM cell lines (n = 3 independent experiments, dots show individual technical repeats). shFGFR1 cells showed significantly reduced cell migration compared to non-targeting controls (shCo), while shFGFR2 cells did not. **(C)** Representative images (inverted grayscale of nuclear stain) of hGBM L2 orthotopic xenografts expressing shCo, shFGFR1 or shFGFR2 vectors. Insets show magnification of boxed areas. Tumors derived from control and shFGFR2 cells are diffusely invasive while shFGFR1-derived tumors are less invasive. Scale bar 5 mm (insets 50 μm). **(D)** Quantification of tumor invasion of hGBM L2 xenografts (n = 3 animals) shows a significant reduction in tumor invasion in shFGFR1-derived tumors, while shFGFR2-derived tumors were significantly more invasive. **(E)** Quantification of tumor area of hGBM L2 xenografts (n = 3 animals) shows shFGFR1-derived tumors are significantly smaller than shCo or shFGFR2 tumors. (n.s. not significant; *p < 0.05, **p < 0.01, ***p < 0.001).

Because invasive GBM cells show differential expression of FGFR1 and FGFR2, we asked whether FGFR1 is functionally relevant for GBM migration/invasion. We performed sphere outgrowth assays *in vitro* and quantified tumor invasion *in vivo*. Knockdown of FGFR1 in patient-derived GBM cell lines reduced cell migration compared to non-targeting controls, whereas FGFR2 knockdown did not affect cell migration (Fig. 3A,B, S1B). Likewise, ablation of FGFR1 resulted in a profound reduction in tumor invasion in xenografted GBM tumors (Fig. 3C and D). Contrastingly to shFGFR1, xenografted shFGFR2 cells showed significantly increased tumor invasion (Fig. 3C and D). In addition to reduced invasion, xenografted tumors derived from shFGFR1 cells were smaller compared to control and shFGFR2 tumors (Fig. 3E). These results are in line with our previous work demonstrating functional relevance of FGFR1 for stemness in GBM and increased invasive propensity of GBM cancer stem cells [9,11,35]. Hence, FGFR1 is functionally relevant for tumor invasion in GBM.

**Fig. 4:**
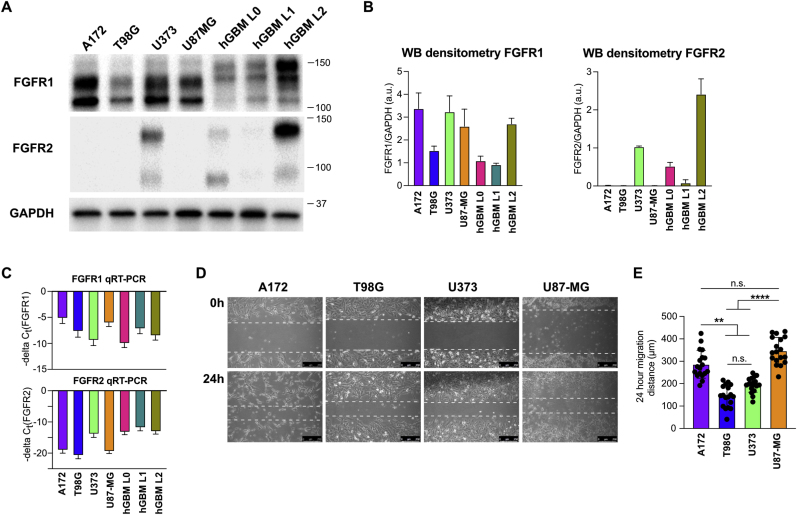
**Comparison of FGFR expression across GBM cell lines. (A)** Western blot showing different expression levels of FGFR1 and FGFR2 across GBM cell lines. Note the differences in FGFR1 isoform expression between cell lines A172, T98G, U373, and U87-MG and primary patient-derived lines hGBM L0, L1, and L2. **(B)** Densitometry of FGFR1 (left) and FGFR2 (right) protein levels (n = 3 independent experiments). **(C)** qPCR quantification of FGFR1 (top) and FGFR2 (bottom) RNA levels, presented as negative delta C_t_ values (lower is less expression; n = 3 independent experiments). **(D)** Representative images from scratch assays of A172, T98G, U373, and U87-MG cells. Dotted lines show migration front at each time point. Scale bars 250 μm. **(E)** Quantification of A172, T98G, U373, and U87-MG cell migration (n = 3 independent experiments). (n.s. not significant; **p < 0.01, ****p < 0.0001).

To further investigate the roles of FGFR1 and FGFR2 in GBM cell migration, we compared FGFR expression levels between primary patient-derived GBM cells (L0, L1, L2) and GBM cell lines A172, T98G, U373, and U87-MG (Fig. 4A). While all tested cell lines expressed FGFR1, the patient-derived GBM cells showed a different pattern of FGFR1 isoforms compared to established cell lines and particularly the presence of a higher molecular weight (∼150 kDa) isoform. Expression of FGFR2 was not detectable in GBM lines A172, T98G, and U87-MG. We further quantified FGFR1 and FGFR2 RNA expression using qPCR (Fig. 4C). This demonstrated that RNA expression patterns for FGFR1 differ from protein expression, with U373 cells showing high FGFR1 protein levels but comparatively low RNA levels. Similarly, in some cell lines where FGFR2 protein was undetectable we observed comparatively high expression of FGR2 RNA (hGBM L1). To evaluate whether endogenous expression levels of FGFR1 or FGFR2 correlated with GBM cell migration, we compared cell migration distances of A172, T98G, U373, and U87-MG cell in a scratch assay (Fig. 4D and E). A172 and U87-MG cells were the most migratory cell lines, with T98G and U373 cells showing significantly less migration. Among the less migratory cells, T98G show the lowest expression levels of FGFR1 and U373 is the only cell line with measurable levels of FGFR2 expression. While the number of cell lines in our analysis is too low for a definitive conclusion, our results are consistent with a pro-migratory role of FGFR1 and an anti-migratory role of FGFR2.

### 3.4 Transcriptional profiling of FGFR1 and FGFR2 knockdown cells identifies gene networks associated with tumor invasion

**Fig. 5:**
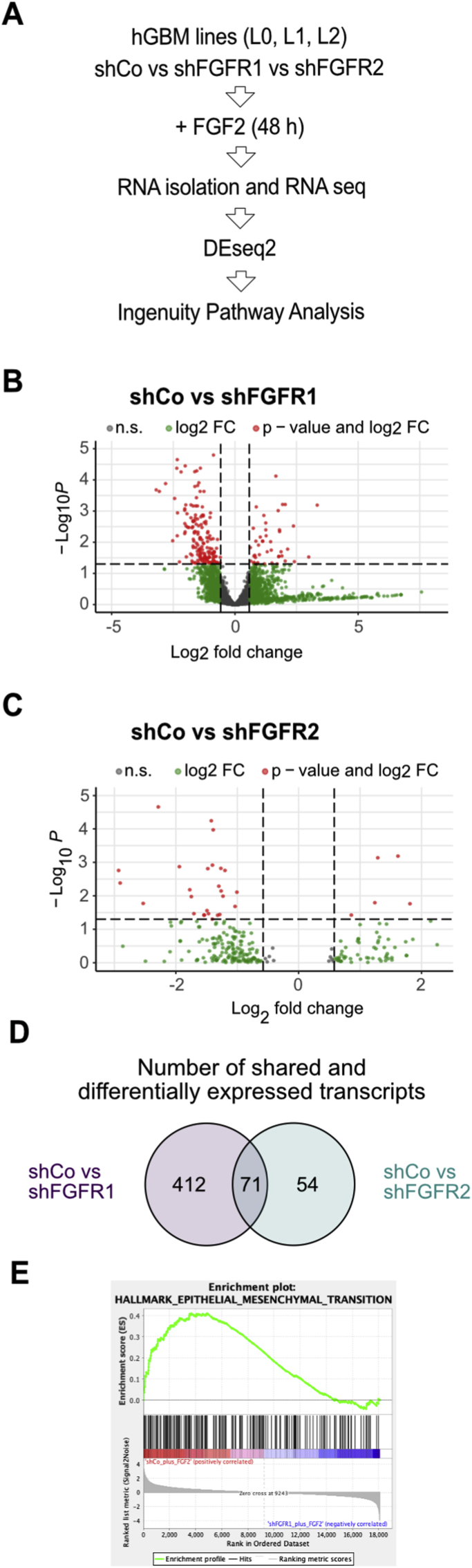
**RNA-seq analysis of shCo, shFGFR1 and shFGFR2 GBM cells. (A)** Schematic of experimental design. Three primary patient derived GBM cell lines were transduced with shCo or shFGFR1 or shFGFR2 vectors. Cells were treated with FGF2 for 48 h prior to RNA isolation and library preparation. DEseq2 was used to identify differentially expressed genes in RNA-seq datasets, which were subsequently analyzed by Ingenuity Pathway Analysis. **(B, C)** Volcano plots showing differentially expressed genes for shCo and shFGFR1 (**B**) and shCo and shFGFR2 (**C**) analysis (n.s. not significant; FC fold change). **(D)** Venn diagram showing overlapping and unique differentially expressed genes between shFGFR1 and shFGFR2 analyses. **(E)** GSEA plot shows enrichment of epithelial-mesenchymal transition signature in shCo compared to shFGFR1 cells (NES = 1.22, p < 0.0001).

To better understand the relevance of FGFR1 for GBM cell migration and invasion and to identify potential gene-regulatory networks involved in tumor invasion of FGFR1-expressing GBM cells, we performed transcriptional profiling using RNA sequencing (RNA-seq). We compared knockdown of FGFR1 and FGFR2 to non-targeting controls across 3 primary patient-derived human GBM cell lines, using single-end RNA-seq (Fig. 5A–C). All cells were treated with FGF2 for 48 h prior to RNA preparation. Differentially expressed genes (DEG) were identified for shFGFR1 versus control and shFGFR2 versus control using DEseq2 [31]. After collapsing genes with multiple transcripts, we found a total of 483 DEG between control and shFGFR1 cells, and a total of 125 DEG between control and shFGFR2 cells (Fig. 5B and C). We compared expression of FGFR1 and FGFR2 transcripts, finding that shFGFR1 samples expressed fewer FGFR1 transcripts and shFGFR2 samples expressed fewer FGFR2 transcripts (Fig. S1C). We then compared DEG between shFGFR1 and shFGFR2 samples, finding that the majority of shFGFR2 DEG (71) were shared with shFGFR1. Contrastingly, over 80% of shFGFR1 DEG (412) were unique to FGFR1 ablation (Fig. 5D). We used gene set enrichment analysis (GSEA) to identify relevant gene sets in shFGFR1 and shFGFR2 samples. This revealed that gene sets for epithelial-mesenchymal transition were significantly enriched in controls compared to shFGFR1 (Fig. 5E), but not shFGFR2 (not shown), validating our previously reported link between FGFR1 and the transcription factor ZEB1 [11].

**Fig. 6:**
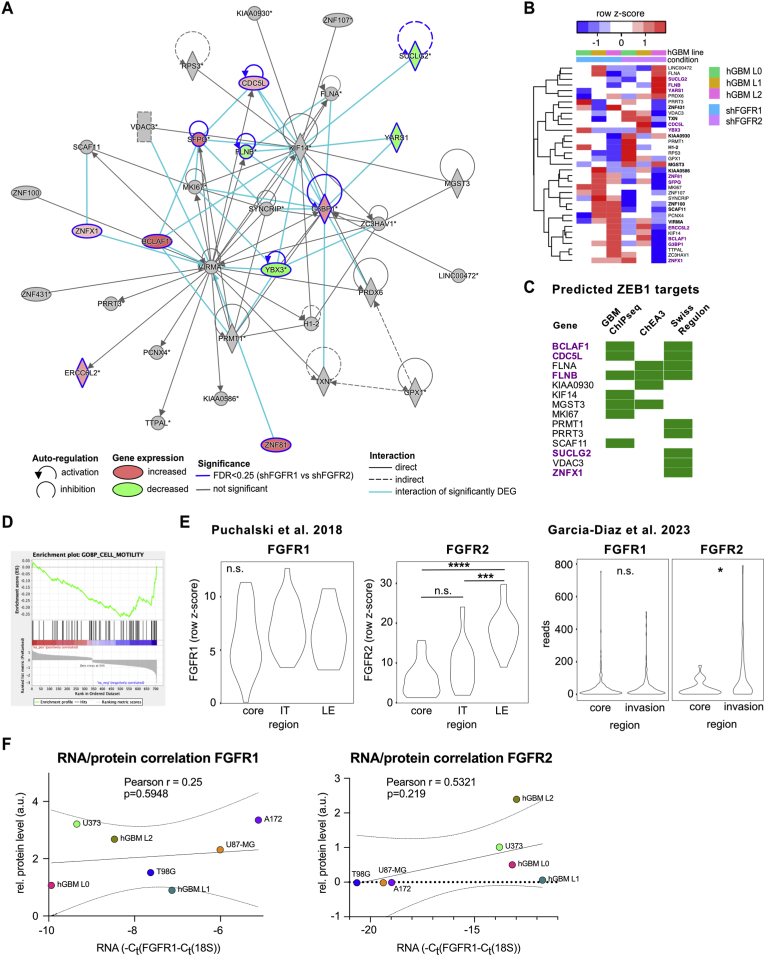
**Network analysis reveals FGFR1-specific gene targets associated with cell motility. (A)** Ingenuity pathways map of cell motility-associated gene network that is regulated by FGFR1. Genes with significant expression differences between shCo and shFGFR1 samples are highlighted in green (decreased expression) or red (increased expression) with color shade indicating the level of change. Genes that are significantly different between shFGFR1 and shFGFR2 analyses are outlined in blue. **(B)** Heatmap showing gene network candidates normalized reads (z-scores) for all samples. Genes with significant expression differences between shCo and shFGFR1 samples are highlighted in bold, genes that are significantly different between shFGFR1 and shFGFR2 analyses are purple. **(C)** ZEB1 target predictions of cell motility network members from 3 separate datasets (see text for details). Only genes with at least one predicted hit are shown. Candidates with significant predictive values from each dataset are highlighted in green. Genes that are significantly different between shFGFR1 and shFGFR2 analyses are purple. **(D)** GSEA plot shows enrichment for cell motility associated genes in shCo compared to shFGFR1 cells (NES = −1.97, p < 0.0001). **(E)** RNA-seq analysis of FGFR1 and FGFR2 expression in public datasets (see text for details) with spatial information. IT = invasive tumor, LE = leading edge (n.s. not significant; *p < 0.05, ***p < 0.001, ****p < 0.0001). **(F)** Pearson correlation for FGFR1 (left) and FGFR2 (right) protein and RNA expression (mean values from n = 3 independent experiments, see Fig. 4). Solid line shows trend with dotted lines depicting 95% confidence intervals.

We then used QIAGEN Ingenuity Pathway Analysis [36] for a comparative analysis of shFGFR1 versus shFGFR2 results. IPA analysis yielded a total of 1564 transcripts in shFGFR1 cells and 1851 transcripts in shFGFR2 cells that passed significance threshold. IPA comparison between FGFR1 and FGFR2 knockdown revealed 56 differentially activated canonical pathways that reached the significance threshold of a Benjamini-Hochberg p value of 0.05 in shFGFR1 cells. Of note, the network ‘RhoA signaling’ showed a negative z-score in shFGFR1 cells (meaning network genes are more likely downregulated) but a positive z-score in shFGFR2 cells (Benjamini-Hochberg p-value 0.006). RhoA signaling is linked to cell shape, locomotion, and polarity, and downregulation of RhoA network genes following loss of FGFR1 supports decreased cell motility and invasion in these cells. A comparison of biological functions in the Qiagen knowledge base showed significant association of ‘Cell movement of tumor cell lines’ and ‘Migration of tumor cell lines’ (both B–H p value 0.003) with shFGFR1, but not shFGFR2 cells. We used Qiagen's functional network analysis to further identify gene networks associated with cell motility and tumor invasion, which identified a network of cell motility-associated gene targets (Fig. 6A and Table 1). Of the candidates in this network, 20 DEG were significantly enriched in shFGFR1 samples compared to control (Fig. 6B). To identify potential candidate gene sets involved in cell migration that are specific to FGFR1 signaling, we curated a list of DEG from shFGFR1 cells that are not significantly changed following FGFR2 knockdown. We further compared network candidates between shFGFR1 and shFGFR2 samples and found 11 of those 20 candidates (BCLAF1, CDC5L, ERCC6L2, FLNB, G3BP1, SFPQ, SUCLG2, YARS1, YBX3, ZNF81, ZNFX1) showing significant changes (Fig. 6B). We have shown that the transcription factor ZEB1 is a downstream regulator of FGFR1 signaling [11]. To identify FGFR1-dependent candidates that are potentially regulated by ZEB1, we compared the list of network genes against a chromatin-immunoprecipitation sequencing dataset of ZEB1 [37], computational transcription factor enrichment analysis [38], and a database of genome-wide annotations of regulatory sites [39]. We found overlap with at least 2 of the 3 datasets for BCLAF1, CDC5L, FLNB, and MGST3. Only FLNB overlapped with all 3 datasets (Fig. 6C). Next, we ranked FGFR1-specific DEGs by expression log fold change and performed gene set enrichment analysis for gene ontologies associated with cell motility. This revealed a significantly lower expression of candidate genes linked associated with GO terms ‘cell motility’, ‘wound healing’, ‘cell adhesion’, ‘taxis’, ‘locomotion’, ‘amoeboidal type cell migration’, and ‘regulation of locomotion’ (FDR q-values 0.021, 0.020, 0.024, 0.033, 0.040, 0.049, 0.049, respectively). An exemplary enrichment plot for ‘cell motility’ is shown in Fig. 6D and candidates are listed in Table 2. Enrichment plots for other GO terms are included as supplementary data (Fig. S2).

**Table 1:** Cell motility-associated gene targets regulated by FGFR1. Table lists candidates from ingenuity pathway analysis with expression p-values and log ratio.

Symbol	Entrez Gene Name	Entrez Gene Symbol	Entrez Gene ID	Expr p-value	Expr FDR (q-value)	Expr Log Ratio
BCLAF1	BCL2 associated transcription factor 1	BCLAF1	9774	0.001953	0.073631	0.864912
CDC5L	cell division cycle 5 like	CDC5L	988	0.012552	0.223788	0.378293
ERCC6L2	ERCC excision repair 6 like 2	ERCC6L2	375748	0.01325	0.231946	0.493005
FLNA	filamin A	FLNA	2316	0.039747	0.401231	−0.76774
FLNB	filamin B	FLNB	2317	0.000277	0.023391	−0.74711
G3BP1	G3BP stress granule assembly factor 1	G3BP1	10146	0.000675	0.039159	0.547916
GPX1	glutathione peroxidase 1	GPX1	2876	0.038261	0.394282	−0.75749
H1-2	H1.2 linker histone, cluster member	HIST1H1C	3006	9.04E-06	0.002792	−1.34326
KIAA0586	KIAA0586	KIAA0586	9786	0.00096	0.049144	0.830086
KIAA0930	KIAA0930	KIAA0930	23313	0.000767	0.042201	−0.51689
KIF14	kinesin family member 14	KIF14	9928	0.027563	0.335247	0.606627
LINC00472	long intergenic non-protein coding RNA 472	LINC00472	79940	0.017333	0.268009	0.743055
MGST3	microsomal glutathione S-transferase 3	MGST3	4259	0.005654	0.143887	−1.06294
MKI67	marker of proliferation Ki-67	MKI67	4288	0.024443	0.315163	0.612561
PCNX4	pecanex 4	PCNX4	64430	0.017013	0.265733	0.532279
PRDX6	peroxiredoxin 6	PRDX6	9588	0.028787	0.342414	−0.41061
PRMT1	protein arginine methyltransferase 1	PRMT1	3276	0.008606	0.18267	−0.6363
PRRT3	proline rich transmembrane protein 3	PRRT3	285368	0.028585	0.340981	0.779732
RPS3	ribosomal protein S3	RPS3	6188	0.039521	0.400175	−0.74104
SCAF11	SR-related CTD associated factor 11	SCAF11	9169	0.004361	0.121373	0.596014
SFPQ	splicing factor proline and glutamine rich	SFPQ	6421	0.006349	0.153251	0.732066
SUCLG2	succinate-CoA ligase GDP-forming subunit beta	SUCLG2	8801	0.001367	0.060977	−0.90376
SYNCRIP	synaptotagmin binding cytoplasmic RNA interacting protein	SYNCRIP	10492	0.015855	0.255438	0.720396
TTPAL	alpha tocopherol transfer protein like	TTPAL	79183	0.017579	0.269711	0.66794
TXN	thioredoxin	TXN	7295	0.011825	0.21552	−1.42027
VDAC3	voltage dependent anion channel 3	VDAC3	7419	0.049224	0.446207	0.406374
VIRMA	vir like m6A methyltransferase associated	KIAA1429	25962	0.010037	0.198421	0.394915
YARS1	tyrosyl-tRNA synthetase 1	YARS	8565	0.002762	0.090311	−0.93341
YBX3	Y-box binding protein 3	YBX3	8531	0.009108	0.189025	−1.03785
ZC3HAV1	zinc finger CCCH-type containing, antiviral 1	ZC3HAV1	56829	0.02123	0.295277	0.447403
ZNF100	zinc finger protein 100	ZNF100	163227	0.000482	0.031522	1.40418
ZNF107	zinc finger protein 107	ZNF107	51427	0.03152	0.358747	0.912181
ZNF431	zinc finger protein 431	ZNF431	170959	0.015702	0.254676	0.850297
ZNF81	zinc finger protein 81	ZNF81	347344	0.006946	0.161442	0.766573
ZNFX1	zinc finger NFX1-type containing 1	ZNFX1	57169	0.014958	0.248574	0.316956

**Table 2:** Gene set enrichment analysis candidates. Table lists candidates from GSEA associated with cell motility with rank and running enrichment score.

Name	Symbol	Rank in Gene List	Rank Metric Score	Running es	Core Enrichment
row_0	TP53INP1	3	1.27764904	0.01921398	No
row_1	CDKN2B-AS1	7	1.13283801	0.03571892	No
row_2	ASPM	41	0.83374202	−2.46E-04	No
row_3	PARVA	67	0.73762798	−0.0255098	No
row_4	DEPDC1B	117	0.62990201	−0.0902885	No
row_5	PARD6B	126	0.615991	−0.0912649	No
row_6	KIF14	132	0.60662699	−0.087729	No
row_7	JAGN1	141	0.59868002	−0.0890293	No
row_8	TBC1D24	145	0.59428799	−0.0825992	No
row_9	BRAF	173	0.559798	−0.1143144	No
row_10	SYDE1	175	0.55852997	−0.1054282	No
row_11	MAZ	178	0.55233002	−0.0982206	No
row_12	PDCD6	198	0.53384399	−0.1179212	No
row_13	ANLN	210	0.51605499	−0.1254547	No
row_14	ARPIN	226	0.49285701	−0.1396721	No
row_15	KANK2	228	0.49211401	−0.1320285	No
row_16	PHACTR4	243	0.47075301	−0.1450969	No
row_17	TAOK2	256	0.445806	−0.155507	No
row_18	CEP131	259	0.43920201	−0.1504157	No
row_19	CRK	280	0.40059701	−0.1741716	No
row_20	DIAPH1	327	0.30548501	−0.2403318	No
row_21	STK4	330	0.30057299	−0.2378338	No
row_22	RAF1	336	0.26874399	−0.2406188	No
row_23	RHOA	339	0.25658399	−0.2389438	No
row_24	AMOTL1	340	0.20852999	−0.2350428	No
row_25	ARRB2	351	−0.30765	−0.2449124	No
row_26	BSG	360	−0.35071	−0.2508516	No
row_27	STK24	364	−0.36667	−0.2486796	No
row_28	CDC42BPB	382	−0.40257	−0.2677111	No
row_29	PLXNA1	383	−0.40391	−0.260155	No
row_30	MYO18A	390	−0.4181	−0.2617084	No
row_31	LGMN	418	−0.48845	−0.2947583	No
row_32	CD151	424	−0.49881	−0.2932394	No
row_33	ARPC3	435	−0.5158	−0.2992151	No
row_34	FUT10	438	−0.52039	−0.292605	No
row_35	SEMA6C	442	−0.52626	−0.2874475	No
row_36	IGSF8	466	−0.57275	−0.3126704	No
row_37	PRKCE	505	−0.64769	−0.3599288	No
row_38	ADD2	506	−0.64852	−0.3477967	No
row_39	CD99	516	−0.66842	−0.3493547	No
row_40	MITF	520	−0.68504	−0.3412269	No
row_41	SPHK1	537	−0.72581	−0.3526489	No
row_42	FLNA	552	−0.76774	−0.3601615	Yes
row_43	PTPRF	557	−0.77338	−0.3519436	Yes
row_44	DNAH1	561	−0.78178	−0.342006	Yes
row_45	MERTK	565	−0.79203	−0.3318767	Yes
row_46	NINJ1	569	−0.80225	−0.3215562	Yes
row_47	CSNK2B	575	−0.82087	−0.3140124	Yes
row_48	KCTD13	576	−0.82126	−0.2986487	Yes
row_49	MDK	589	−0.86355	−0.301244	Yes
row_50	SEMA4C	590	−0.86373	−0.2850859	Yes
row_51	TTC21A	595	−0.87663	−0.2749364	Yes
row_52	MAPK15	596	−0.87707	−0.2585287	Yes
row_53	PLCG2	600	−0.88218	−0.2467129	Yes
row_54	PTP4A3	605	−0.89508	−0.2362183	Yes
row_55	SERPINF1	607	−0.90402	−0.220869	Yes
row_56	LAMC3	630	−0.97518	−0.2370009	Yes
row_57	SERPINE2	649	−1.07105	−0.2450893	Yes
row_58	SDC4	677	−1.31201	−0.2627325	Yes
row_59	HBEGF	679	−1.31889	−0.2396221	Yes
row_60	GADD45A	681	−1.3538899	−0.2158568	Yes
row_61	TRIB1	691	−1.52238	−0.2014396	Yes
row_62	DDIT4	692	−1.5403399	−0.1726238	Yes
row_63	CARMIL3	693	−1.56016	−0.1434373	Yes
row_64	FGFR1	694	−1.58595	−0.1137683	Yes
row_65	PLAU	699	−1.79008	−0.0865306	Yes
row_66	DDIT3	704	−2.0682001	−0.05409	Yes
row_67	SERPINE1	706	−3.0584099	0.00156241	Yes

To further validate differences in FGFR1 and FGFR2 expression within GBM, we identified publicly available RNA-seq datasets that compared tumor core versus infiltrating regions. We used a dataset based on bulk RNA-seq of histologically defined regions within patient samples [34] and another dataset based on single cell RNA-seq of microdissected regions in somatic mouse models of GBM [40]. In both datasets, we found no difference in FGFR1 expression between tumor core and infiltrating regions, consistent with our own data. Surprisingly, both datasets showed significantly increased FGFR2 expression in infiltrating regions (Fig. 6E), which contrasted with our findings. Because RNA-seq data is based on gene expression, but our observations are based on FGFR2 protein levels and because we found differences in RNA and protein expression for FGFR1 and FGFR2 in some of the GBM lines (Fig. 4B and C), we decided to test the correlation between FGFR RNA and protein levels in our samples. A Pearson correlation showed that there is no relationship between FGFR1 or FGFR2 RNA and protein levels (Fig. 6F). While our data relies on *in vitro* expression of RNA and protein in cell lines, it supports that FGFR protein levels can deviate substantially from RNA expression and that FGFRs may be subject to post-translational regulation.

Together, our results strongly support that FGFR1, but not FGFR2, promotes tumor invasion in GBM, linking spatial distribution of receptor expression within GBM to function on cell motility. We identify relevant gene sets associated with cell motility that are regulated by FGFR1.

## 4 Discussion

Among the four main FGFRs, expression levels of FGFR1 and FGFR2 in GBM show a significant but divergent association with patient survival. High expression levels of FGFR1 are linked to poor survival and FGFR1 is a regulator of cancer stem cells and therapy resistance in GBM [11,28,29]. By contrast, the functions of FGFR2 in GBM remain incompletely understood. High expression levels of FGFR2 are associated with increased survival, and downregulation of FGFR2 is correlated with increased proliferation [24]. In addition, FGFR3 expression has been associated with infiltrating GBM cells in a single-cell RNA-seq study [41], and FGFR4 overexpression promoted GBM invasiveness [42].

Here, we investigated the distribution and functional relevance of FGFR1 and FGFR2 in GBM primary patient-derived cell lines and xenografts. We found FGFR1 expressed in tumor mass and infiltrating GBM cells, while FGFR2 expression is confined to the tumor mass and notably absent in diffusely invasive cells. We previously reported that FGFR1 but not FGFR2 or FGFR3 regulated GBM cell proliferation *in vitro* [11]. Thus, the potential functions of FGFR2 within the tumor mass remain unclear and need further investigation. The differential expression of FGFR1/FGFR2 in invasive GBM cells is reflected in the functional consequences of FGFR1 and FGFR2 knockdown, as only ablation of FGFR1, but not of FGFR2, reduced GBM cell migration and tumor invasion.

FGFR1 expression and/or amplification has been linked to epithelial-mesenchymal transition and metastasis in other cancers, including lung cancer, bladder and urothelial cancer, and gastric cancer [[43], [44], [45], [46]]. Of note, a previous study demonstrated that isoform switching of FGFR1 promotes tumor invasion [47]. We have not tested whether different FGFR1 isoforms are expressed in the tumor mass versus invasive cells, and this warrants further investigation. Notably, we found differential expression of FGFR1 isoforms between GBM cell lines A172, T98G, U373, U87-MG and the patient-derived cell lines L0, L1, L2. In GBM, FGFR1 is known to regulate cancer stemness and therapy resistance through the transcription factor ZEB1 [11,28,29], which is also regulating GBM invasion [9]. To identify gene regulatory networks related to FGFR1-driven GBM tumor invasion, we compared transcriptomes of control, FGFR1 knockdown, and FGFR2 knockdown GBM cells using RNA-Seq. We identified transcripts specifically regulated by FGFR1 and not FGFR2 following stimulation with FGF2. We used IPA to identify cell motility-associated gene regulatory networks that are dependent on FGFR1. This revealed a network consisting of 35 candidates and the key node G3BP1, which is significantly higher connected within this network, shows differential expression between control and FGFR1 knockdown samples, and is a predicted target of ZEB1. We further identified 11 candidates in this network that are exclusively regulated by FGFR1, 5 of which (BCLAF1, CDC5L, FLNB, SUCLG2, ZNFX1) are predicted ZEB1 targets. Hence, these candidates may constitute relevant targets to disrupt GBM invasiveness. To our knowledge, these molecules have not been investigated in the context of GBM invasion. G3BP1 is associated with stress granule assembly and G3BP1 knockdown sensitized U87-MG cells to proteasome inhibition [48]. BCLAF1 acts in a positive feedback loop enhancing PDGFRa and EGFR signaling in high-grade glioma [49]. CDC5L expression is associated with tumor progression in glioma [50].

Our study does not resolve whether FGFR1 and FGFR2 show similar spatial distribution in human patients. We analyzed publicly available RNA-seq datasets of human and murine GBM and found in both a significant upregulation of FGFR2 RNA in tumor invasion zones. Notably, there was no correlation between FGFR1 or FGFR2 RNA and protein in the cell lines used in this study, but whether this is an artifact of *in vitro* culture or a general feature of FGFRs remains unclear. Future studies should investigate FGFR protein expression across different histopathological hallmarks in human patient specimens.

In summary, our study demonstrates differential expression of FGFR1 and FGFR2 on invasive GBM cells and provides functional and transcriptomic evidence that FGFR1 regulates tumor invasion in GBM. We found candidate gene targets at the center of a regulator network associated with cell motility that warrant further investigation.

## Data availability

RNA sequencing datasets from control and FGFR1 or FGFR2 knockdown GBM cells have been deposited in ArrayExpress (E-MTAB-13161). Other datasets analyzed in this study are available at the Allen Brain Institute (glioblastoma.alleninstitute.org) [34] and at NCBI’s gene expression omnibus (GSE174470).

## CRediT authorship contribution statement

**Nawal Alshahrany:** Data curation, Formal analysis, Investigation, Writing – original draft. **Ayesha Begum:** Formal analysis, Investigation. **Dorit Siebzehnrubl:** Investigation. **Ana Jimenez-Pascual:** Methodology, Supervision, Writing – review & editing. **Florian A. Siebzehnrubl:** Funding acquisition, Methodology, Supervision, Writing – original draft, Writing – review & editing.

## Declaration of competing interest

The authors declare that they have no known competing financial interests or personal relationships that could have appeared to influence the work reported in this paper.

## Figures and Tables

**figs1:**
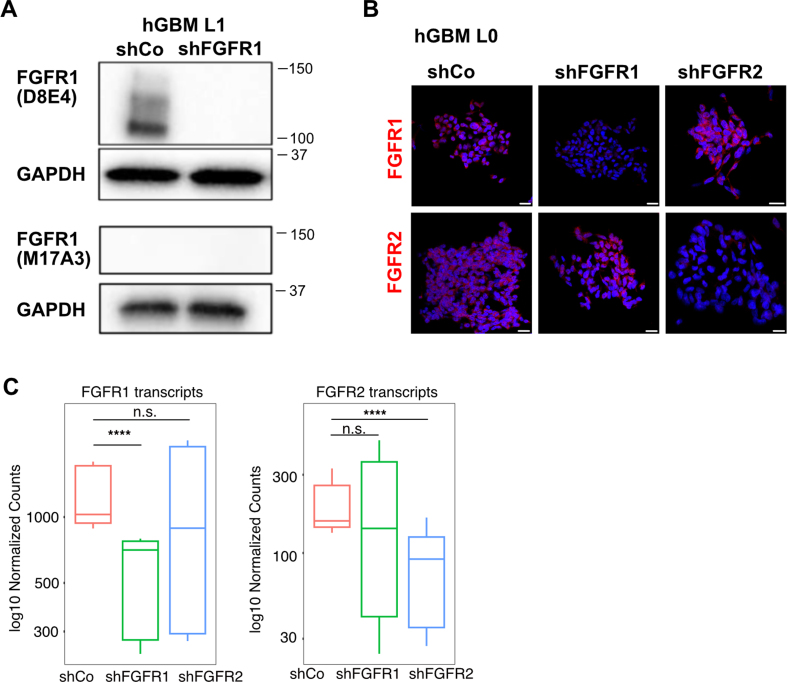


**figs2:**