# Supplementary material for: Spatial distribution and functional relevance of FGFR1 and FGFR2 expression for glioblastoma tumor invasion
Source: Cancer Lett. 2023 Sep 1;571:216349. doi: 10.1016/j.canlet.2023.216349 (PMC10840508; doi:10.1016/j.canlet.2023.216349)
Supplement: Multimedia component 1 [file mmc1.docx]

| **Antibody** | **Species** | **Dilution** | **Manufacturer** |
| --- | --- | --- | --- |
| FGFR2 (D4L2V) | Rabbit IgG | 1:400 | Cell signalling technology |
| FGFR1 (M17A3) | Mouse IgG2b | 1:100 | Novus biologicals |
| FGFR1 (D8E4) | Rabbit IgG | 1:400 | Cell signalling technology |
| Nestin (NES7847983) | Mouse IgG | 1:1000 | Aves lab, inc. |
| Vimentin (CPCA-Vim) | Chicken IgY | 1:5000 | Encor biotechnology inc. |
| GAPDH (HRP conjugated) | Rabbit IgG | 1:10000 | Bio Rad |
| Alexa 488 (A11039) | Goat anti-chicken IgY | 1:500 | Thermo Fisher Scientific |
| Alexa 488 (A21202) | Donkey anti-mouse IgG | 1:500 | Thermo Fisher Scientific |
| Alexa 594 (A21207) | Donkey anti-rabbit IgG | 1:500 | Thermo Fisher Scientific |
| Alexa 647 (A21449) | Goat anti-chicken IgY | 1:500 | Thermo Fisher Scientific |
| Alexa 647 (A31573) | Donkey anti-rabbit IgG | 1:500 | Life technologies |
